# Congenital Syphilis—An Illustrative Review

**DOI:** 10.3390/children10081310

**Published:** 2023-07-29

**Authors:** Deepika Sankaran, Elizabeth Partridge, Satyan Lakshminrusimha

**Affiliations:** 1 Division of Neonatology, Department of Pediatrics, University of California Davis, Sacramento, CA 95817, USA; dsankaran@ucdavis.edu; 2 Division of Infectious Diseases, Department of Pediatrics, University of California Davis, Sacramento, CA 95817, USA; epartridge@ucdavis.edu

**Keywords:** syphilis, congenital syphilis, vertical transmission, treponema pallidum, hydrops

## Abstract

Congenital syphilis is caused by the spirochete, *Treponema pallidum*, which can be transmitted from an infected mother to her fetus during pregnancy or by contact with a maternal lesion at the time of delivery. The incidence of congenital syphilis is rapidly increasing all over world with 700,000 to 1.5 million cases reported annually between 2016 and 2023. Despite the widespread availability of Penicillin, 2677 cases were reported in 2021 in the US. Clinical manifestations at birth can vary widely ranging from asymptomatic infection to stillbirth or neonatal death. Low birth weight, rash, hepatosplenomegaly, osteolytic bone lesions, pseudoparalysis, central nervous system infection, and long-term disabilities have been reported in newborns with congenital syphilis. Prevention of congenital syphilis is multifaceted and involves routine antenatal screening, timely treatment of perinatal syphilis with penicillin, partner tracing and treatment, and health education programs emphasizing safe sex practices and strategies to curb illicit drug use. Neonatal management includes risk stratification based on maternal syphilis history, evaluation (nontreponemal testing, complete blood counts, cerebrospinal fluid, and long-bone analysis), treatment with penicillin, and followup treponemal testing. Public health measures that enhance early detection during pregnancy and treatment with penicillin, especially in high-risk mothers, are urgently needed to prevent future cases of congenital syphilis.

## 1. Introduction

Syphilis is an infectious disease caused by the bacterium *Treponema pallidum* subspecies *pallidum*. This sexually transmitted infection (STI) is acquired horizontally by contact with an infected skin lesion (chancre, mucus patch, or condyloma latum). *T. pallidum* can also be acquired vertically, from an infected mother to fetus by transplacental transmission or contact with a maternal lesion during delivery [1]. Once thought to be eradicated, this organism has reemerged as a global pathogen [2]. Globally, perinatal syphilis is the second leading cause of stillbirths, and is associated with significant morbidity and mortality among affected newborns with congenital syphilis [3]. Although some countries such as Cuba and Thailand have successfully eliminated congenital syphilis, the rates are rising in many other countries [4,5]. Timely pre- and perinatal screening followed by adequate treatment of maternal syphilis infection using penicillin can prevent the development of congenital syphilis [6].

## 2. Epidemiology of Syphilis

Syphilis was first reported in the late 15th century as a new disease that affected French soldiers. After aggressively spreading through Europe as “the Great pox”, the clinical features evolved from severe (painful sores and significant mortality) to milder symptoms over the next 50 years, likely due to natural selection of less virulent strains [7]. Girolamo Fracastoro, a poet and scientist, wrote about a shepherd named Syphilus who angered the god Apollo who then cursed people with a disease named “syphilis”, after the shepherd’s name [8]. The causative agent *Treponema pallidum* (Trepo—to turn, nema—thread in Greek) subspecies *pallidum* was identified in 1905 by Schaudinn and Hoffman, 4 centuries after the first report of this disease. Following the advent of Penicillin, there was a 75% reduction in incidence of syphilis in the US by 1954 compared to 1944 followed by a 90% reduction in 1975. There was a corresponding reduction in rates of congenital syphilis from 368/100,000 to 37/100,000 [9]. By the end of 1990s, syphilis incidence was on the decline globally and reached 2.1 cases per 100,000 persons [10]. This decrease was attributed primarily to the control of infection and reduction in mortality after widespread use of penicillin and in part to safer sexual behavior prompted by the AIDS epidemic. However, despite the national syphilis elimination effort in the US by the Centers of Disease Control and Prevention (CDC), the rates of syphilis increased. In 2013, the elimination plan was terminated due to lack of funding coinciding with the sharp increase in the number of new cases [11]. In the US, incidence of primary and secondary syphilis in women aged 15–44 increased 52.3% between 2020 and 2021. Correspondingly, the incidence of congenital syphilis increased by 30.5% in 2021 relative to 2020, which is an increase of 219.3% relative to 2017 [12]. The national rate of congenital syphilis incidence in the US is 77.9 cases per 100,000 live births [12]. 

The World Health Organization (WHO) advocates for antenatal screening programs to target global elimination of maternal-to-child transmission of syphilis and occurrence of congenital syphilis. The 2007 initiative by WHO recommended political commitment and advocacy, increasing access to and quality of maternal and newborn health services, screening of all pregnant persons and treatment of all positive cases and their partners and establishing an underlying foundation of surveillance, monitoring, and evaluation [13]. WHO’s global targets included the following: (i) At least 90% of pregnant people are tested for syphilis; (ii) At least 90% of seropositive pregnant people receive adequate treatment by 2015 [14]. However, neither of these targets were met. In a recent WHO Global progress report, 7.1 million adults were newly infected with syphilis in 2020 [14]. The WHO also reported that “congenital syphilis is the second leading cause of preventable stillbirth globally” and that the WHO is “working towards eliminating mother-to-child transmission of HIV, syphilis and hepatitis B” [14]. Although some countries such as Cuba, Thailand, Malaysia, Maldives, and Sri Lanka have successfully eliminated congenital syphilis, rates have been rising in most low-to-middle income countries (LMICs) and in some high-income countries (HICs) such as the US, Greece, Japan, and the United Kingdom [4]. 

Although transmission of syphilis is more prevalent in at risk populations marked by substance abuse, poor socioeconomic status, and disadvantaged people, it is increasing globally in low-risk populations [15]. The risk factors that contribute to increases in the incidence and prevalence of syphilis transmission in the community may vary between HICs and LMICs. 

### 2.1. Factors Associated with the Increase in Congenital Syphilis in the US

In the US, there has been a sevenfold increase in the number of congenital syphilis cases between 2012 and 2021. This increase does not uniformly affect geographical areas, races, and ethnicities. In 2020, approximately half of the congenital syphilis cases reported were from California and Texas [16]. Incidence rates of congenital syphilis increased approximately threefold for persons of color (threefold for Black and three-point-five-fold for Hispanic/Latinx) between 2016 and 2020. These racial disparities reflect longstanding racial inequalities in the US. Congenital syphilis disproportionately affects patients in non-English-speaking households, with lower socioeconomic status, low maternal education levels, high-risk sexual behaviors, substance abuse (especially methamphetamine and heroine use), unstable housing, domestic violence, lack of health insurance, and poor access to prenatal care [15]. Furthermore, stigma or fear associated with substance use and mental health issues have been reported as barriers to seek medical assistance for timely testing and treatment of syphilis in pregnant people [17].

The increased rate of congenital syphilis in the US is likely linked to the 40% decrease in federal funding for STI prevention programs since 2000 [15,18,19]. These programs provide STI education, testing, contact tracing, and treatment, all of which are essential to the prevention of congenital syphilis. A 2018 Morbidity and Mortality Weekly report identified a lack of appropriate treatment of maternal perinatal syphilis after timely diagnosis in 31% of the mothers [20]. Additional missed opportunities included a lack of timely prenatal care in 28% and late identification of seroconversion in 11% [20]. 

### 2.2. Factors Associated with the Global Increase in Congenital Syphilis Infection

Approximately 90% of all syphilis infections are estimated to occur in LMICs with the highest burden in African countries which carry 62% of the global congenital syphilis cases [4,21,22]. Although bearing a significant burden of congenital syphilis, there are limited data on risk factors in African countries [4]. LMICs have a disproportionately high burden of other STIs such as HIV [23]. In an observational study spanning 1990–2019, Sub-Saharan Africa and Latin America had the highest age-standardized incidence rate of STIs in 2019 with an uptrend in syphilis cases between 2010 and 2019 [24,25] transmission. A recent systematic review that investigated the costs of scaling up HIV and syphilis testing in LMICs, revealed testing uptake and kits as the major drivers of the costs. Eliminating mother-to-child transmission of syphilis through expanded screening and treatment during antenatal or prenatal care is likely to be cost effective globally and can result in a reduction in the incidence of congenital syphilis [26,27]. Future research is warranted to study the factors influencing increasing syphilis rates in LMICs [4,28]. In addition to identifying factors involved, commitment from the countries to develop policies, testing, and followup guidelines and mitigation strategies are needed to reduce transmission of syphilis during pregnancy. Adequate funding to support antenatal screening and testing including rapid point-of-care testing in remote/rural settings and appropriate health information systems to report the results to public health authorities may also need to be considered [13]. Moreover, the case definition used for surveillance of congenital syphilis varies among different countries making the interpretation of prevalence data challenging [4].

In China, following the development of a national plan to prevent maternal-to-child transmission of syphilis in 2011 that was promoted in 2015, there was a reduction in the incidence of congenital syphilis from 91.6 cases per 100,000 livebirths in 2011 to 11.9 cases per 100,000 livebirths in 2019 [4,29]. In spite of this decrease nationally, due to existing economic (migrant workers), cultural, and ethnic differences in a vast country, there are variations in disease distribution between regions [30,31]. In a prospective cohort study from Shenzen, South China, maternal education, history of syphilis, paternal age, and education were negatively associated with congenital syphilis [32]. On the contrary, maternal unmarried status, inadequate antenatal care, more sexual partners, every week delay in treatment, higher baseline titers of nontreponemal antibodies, early syphilis, nontreatment with penicillin, and paternal cocaine use increased risk of congenital syphilis [33]. More recently, China has seen an increase in the incidence of congenital syphilis, probably secondary to a wide range of sociobiological factors [30,33].

Socioeconomic factors, cultural social, and territorial differences play a significant role in the differences in the incidence of congenital syphilis that is observed between countries despite similar strategies to control the transmission and management of syphilis [30,34]. Among the countries reporting to the Pan-American Health Organization, Brazil reported the majority of the congenital syphilis cases in 2015 [4]. Brazil also did not reach the congenital syphilis elimination goal of ≤0.5 cases per 1000 live births by 2015 [4,35]. Subsequently, in 2015 The Brazilian Ministry of Health established the “Syphilis No!” project and enhanced the program on “Applied Research for Intelligent Integration Aimed at Strengthening Healthcare networks for Rapid Response to Syphilis”. These efforts aimed to reduce acquired syphilis, perinatal syphilis, and congenital syphilis by expanding diagnostic testing and timely treatment of pregnant persons and partners [35]. This project influenced the syphilis epidemic trends in Brazil from 2018 with a reduction in number of congenital cases in the 100 priority municipalities [35]. The experience from Brazil stresses the importance of streamlining case notification flow via the integration of surveillance, case notification to public health departments, and timely treatment [34]. 

Although the incidence of congenital syphilis is declining in Europe, high prevalence is reported in vulnerable populations with a higher proportion of persons using drugs and transactional sex along with insufficient coverage by preventative services [4,36]. The recent increase in congenital syphilis cases in HICs such as Canada (more than doubled between 2016 and 2020) reflects the inherent barriers to healthcare access to prenatal care, testing, and treatment that require urgent attention from public health authorities [4,37]. Moreover, a disproportionate increase in the incidence of congenital syphilis was observed in children born to Asian and Indigenous women in Canada [38]. 

## 3. Perinatal Syphilis—Clinical Features, Diagnosis, and Management

Syphilis is transmitted through direct contact with lesions in the skin and mucous membranes such as chancre or hypertrophic papular skin lesions known as condylomata lata [39,40]. Syphilis can be classified into four stages, primary, secondary, latent, and tertiary. The clinical features of perinatal syphilis are the same as those experienced by nonpregnant individuals. However, the symptoms may not be readily attributed to perinatal syphilis unless there is a high index of suspicion [39]. A thorough review of the maternal medical, obstetrical, and social history is crucial in early recognition of risk factors of perinatal syphilis infection [4]. 

### 3.1. Clinical Features

**Figure 1:**
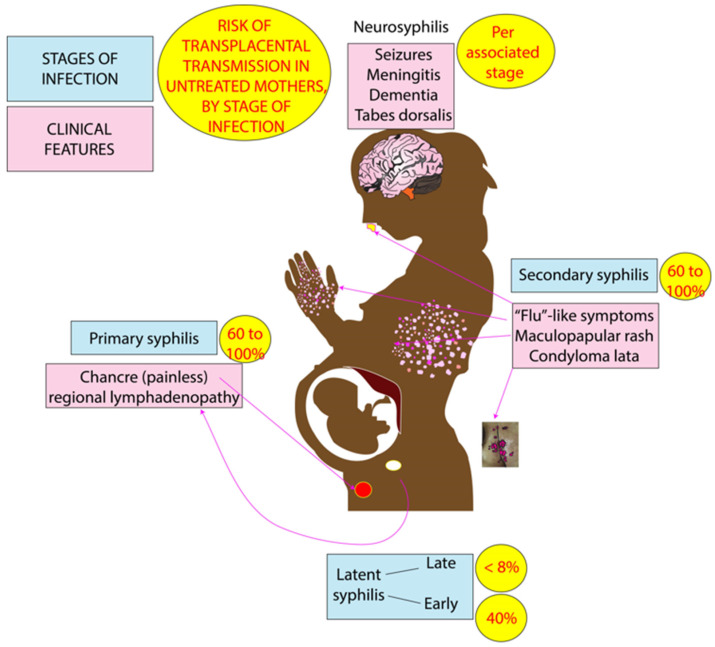
Clinical features of perinatal syphilis and risk of maternal-to-fetal transmission. Primary syphilis (painless skin lesions/chancre or regional lymphadenopathy) and secondary syphilis (flu-like syndrome, maculopapular rash, and skin lesions known as condyloma lata) are associated with 60–100% transmission of infection to the fetus. Latent syphilis with disappearance of symptoms is associated with <8% to 40% risk of transplacental transmission of syphilis infection to the fetus. Neurosyphilis can develop at any stage of infection and present as seizures, meningitis, dementia, or tabes dorsalis. Copyright: Satyan Lakshminrusimha.

Syphilis is often referred to as “the great masquerader”. Due to its versatile clinical presentations, recognition of syphilis infection can be a challenge even for the most experienced clinicians. Furthermore, the natural history of syphilis, whether treated or untreated, can be quite unpredictable. Primary syphilis infection manifests when the *T. pallidum* enters dermal microabrasions or intact mucosal surfaces [39]. This leads to the development of chancre at the site of inoculation and enlarged adjacent lymph nodes between 10–90 days after exposure (Figure 1). Within a few weeks, the symptoms of primary infection spontaneously resolve without treatment. The painless nature of the skin lesion, minimal or no discomfort from the lymphadenopathy and spontaneous resolution of symptoms can delay diagnosis of primary syphilis. Untreated primary syphilis progresses to a flu-like syndrome referred to as secondary syphilis characterized by headache, sore throat, myalgia, arthralgia, and generalized lymphadenopathy [7]. Secondary syphilis may also manifest as a generalized maculopapular rash specifically involving the palms and soles in addition to condylomata lata in the perineum and axilla (typically in warm and moist areas). If secondary syphilis remains untreated, the infection moves into a latent phase during which time all the symptoms resolve [41]. Latent syphilis is classified as “early latent” if the initial syphilis infection occurred within the preceding 1 year or “late latent” if the initial syphilis infection occurred more than 1 year prior or the time of infection is unknown [39,40]. The disappearance of clinical symptoms and signs may mislead both the persons with infection and their partners to believe that they are no longer infectious [17]. On the contrary, if the person with infection remains untreated, the infection may progress towards tertiary syphilis with tissue and bone destruction “gumma” or aortitis that can occur 1–3 decades after the initial infection [39]. Neurosyphilis can develop at any stage of syphilis (primary, secondary, latent, and tertiary) and can cause meningitis, seizures, dementia, and tabes dorsalis [39]. Syphilis can also involve the ears and eyes causing hearing loss, keratitis, or optic atrophy [39]. 

### 3.2. Maternal-to-Fetal Transmission of Syphilis

Maternal-to-fetal transmission of syphilis infection can occur in utero at any trimester via transplacental transmission or during delivery via direct contact with an infected lesion [39]. The highest rates of transplacental transmission are observed in untreated mothers with primary or secondary syphilis in the third trimester of pregnancy (60–100%) compared to mothers with early-latent (40%) or late-latent (<8%) stages (Figure 1) [39]. With the resurgence of syphilis, the use of “SCORTCH” starting with “S” for syphilis can be used as a mnemonic in the screening for intrauterine infections (SCORTCH—syphilis, cytomegalovirus, “others”, rubella, toxoplasmosis, chicken pox, and herpes simplex) [1]. *T. pallidum* is not known to be secreted in breast milk [42]. There is a possibility of transmission of syphilis from direct contact with sores or skin lesions involving the nipple, areola, or breast tissue during breastfeeding or breast milk expression [43]. Breastfeeding is not contraindicated in perinatal syphilis and is considered safe in the absence of a lesion on the breast [44].

### 3.3. Screening (Testing) and Management of Perinatal Syphilis

Timely screening during pregnancy allows early diagnosis of perinatal syphilis. In areas with high prevalence of congenital syphilis or in pregnant persons with risk factors for perinatal syphilis (described above), testing at three time-points during pregnancy is recommended: at the first prenatal visit, in the third trimester, and at delivery [45]. Timely testing is critical because treatment of maternal perinatal syphilis in the early stages is 98% effective in preventing congenital syphilis in the newborn [27,46]. 

Syphilis screening for pregnant persons involves a combination of treponemal and nontreponemal testing. Treponemal tests such as *T. pallidum* particle agglutination (TP-PA), fluorescent treponemal antibody absorption test (FTA-ABS), *T. pallidum* enzyme immunoassay (TP-EIA), and *T. pallidum* chemiluminescence assay (TP-CIA) are serological tests used to detect antibodies against *T. pallidum*. These tests remain positive even after treatment in a majority (75–85%) of persons, and a negative test indicates the lack of infection [6]. Nontreponemal tests such as the Venereal Disease Research Laboratory (VDRL) and Rapid Plasma Reagin (RPR) detect the antibodies against biomarkers that are released from host cells following cellular damage induced by *T. pallidum*. Nontreponemal tests are complicated by high false positive rates especially in persons with connective tissue disorders, advanced age, lymphoma, and in infections such as Epstein–Barr virus, hepatitis, HIV, tuberculosis, malaria, and measles [39]. These nontreponemal tests are used to monitor the response to treatment both in perinatal and congenital syphilis. Adequate response to treatment is indicated by a fourfold decrease in RPR titer within a year. In most individuals, RPR will ultimately become nonreactive several months after effective treatment but in some individuals may remain reactive with low titers (“serofast”) for years [12]. Pregnant persons who are seropositive are infected unless they have documentation of adequate treatment with appropriate serologic response to treatment and RPR titers are low (RPR < 1:4) and stable [39]. Persons with up-trending or persistent high antibody titers may indicate reinfection [39]. 

Comprehensive, nonjudgmental care is encouraged for perinatal syphilis. Maternal treatment involves either a single dose of intramuscular (IM) penicillin (2.4 million units benzathine penicillin G) in the early stages of infection (primary, secondary, or early latent) or three weekly doses of penicillin for late latent or tertiary syphilis. Neurosyphilis warrants 10–14 days of intravenous (IV) aqueous penicillin. Pregnant persons who are allergic to penicillin should undergo desensitization and subsequent treatment with penicillin due to lack of alternate therapy [47]. Contact tracing and treatment of partners is crucial in preventing reinfection in the pregnant person. Untreated STIs are associated with infertility, debilitating illness, increased risk of HIV transmission, and death. Per the WHO, untreated syphilis during pregnancy can result in adverse birth outcomes in 50–80% of cases [14]. 

## 4. Congenital Syphilis: Clinical Features, Diagnosis, and Management

Syphilis transmission from a pregnant person to her fetus can occur at any point during pregnancy or childbirth and results in congenital syphilis [1,41]. 

### 4.1. Clinical Features and Classification

**Figure 2:**
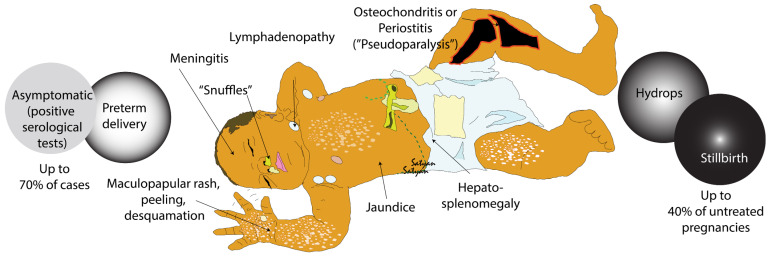
Clinical features of congenital syphilis. A newborn with congenital syphilis may remain asymptomatic in up to 70% of cases, and may be born preterm. Clinical symptoms and signs include snuffles (nasal congestion), maculopapular, peeling, desquamating rash especially involving the palms and soles, lymphadenopathy, jaundice, hepatosplenomegaly, and osteochondritis or periostitis (“pseudoparalysis” due to limited range of movement of affected extremity). Severely infected fetuses may have hydrops fetalis or result in stillbirth (in up to 40% of untreated pregnancies). Copyright: Satyan Lakshminrusimha.

Congenital syphilis infection is classified as early or late based on the onset of clinical symptoms. Large, thick, and pale placenta and abscess-like foci of necrosis in Wharton’s jelly centered around the umbilical vessels (necrotizing funisitis with “barber-pole appearance”) can be observed in congenital syphilis. Early congenital syphilis presents within the first 2 years after birth but most commonly within 3 months after delivery. The symptoms of early congenital syphilis range from asymptomatic (approximately 70% of cases) to stillbirth (up to 40% in untreated pregnancies) and include hydrops fetalis and preterm delivery (Figure 2) [39]. Other symptoms of congenital syphilis are pneumonia, “snuffles” or nasal congestion and excessive nasal discharge, hepatosplenomegaly, lymphadenopathy, hyperbilirubinemia, cholestasis, maculopapular, and desquamating rash that can involve the palms and soles (Figure 2). Central nervous system involvement can present with meningitis and/or seizures [6]. Infants with congenital syphilis can have skeletal system involvement in the form of osteochondritis or periostitis, with or without pseudoparalysis of Parrot (decreased range of movement due to painful periostitis) [12]. Hypoxic ischemic encephalopathy, persistent pulmonary hypertension of the newborn and disseminated intravascular coagulation have also been reported [48]. 

Untreated asymptomatic infants with congenital syphilis infection may present with clinical features after 2 years of age resulting in late congenital syphilis [39]. The clinical presentation at this stage includes the triad of congenital syphilis: interstitial keratitis, sensorineural hearing loss, and notched central incisors or Hutchison teeth. Furthermore, they may develop anterior bowing of the shin (saber shin), painless swelling of the knee (Clutton joints), mulberry molars (rudimentary cusps in first molars), saddle nose, (rarely) uveitis, optic atrophy, and developmental delays [6]. Renal disease in the form of glomerulonephritis and nephrotic syndrome have been reported as well [49]. 

### 4.2. Diagnosis and Management (Figure 3, Table 1)

**Figure 3:**
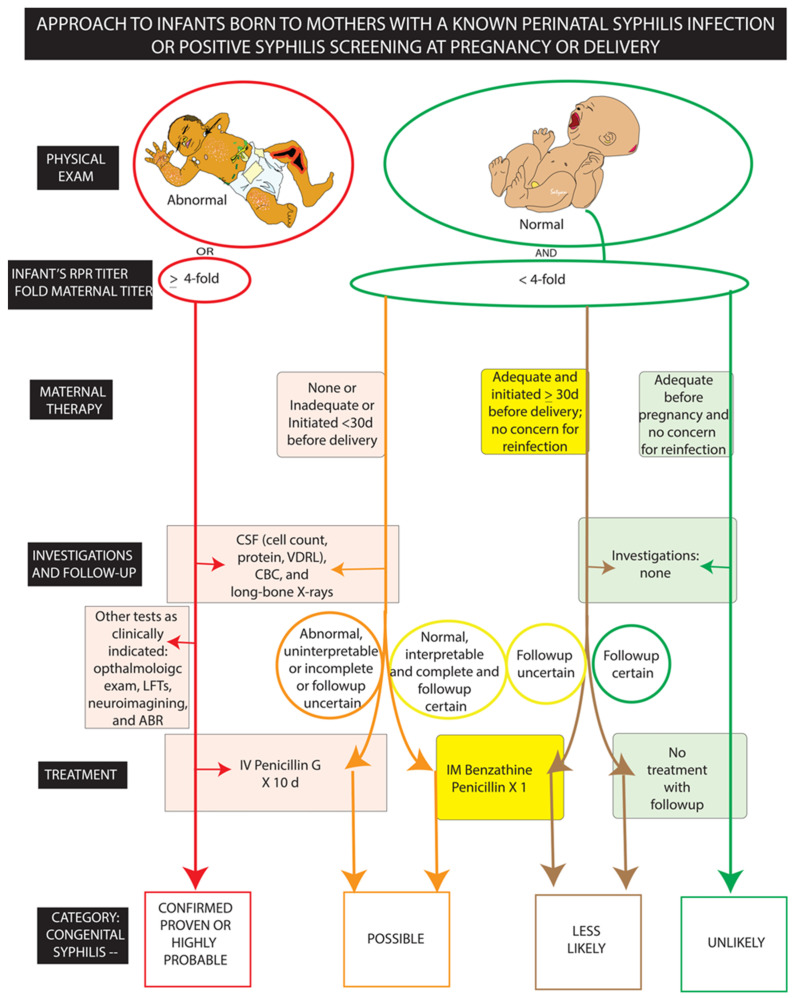
Screening and management algorithm for infants with possible congenital syphilis. CSF, cerebrospinal fluid. RPR, rapid plasma reagin. CBC, complete blood counts. LFT, liver function tests. ABR, auditory brainstem response for hearing test. Infographic based on CDC and AAP guidelines for screening and management of congenital syphilis. Copyright: Satyan Lakshminrusimha.

The evaluation and management of infants and children for congenital syphilis is based on maternal information (history of syphilis infection and treatment, RPR results during pregnancy and at time of delivery, and risk factors for infection/reinfection), infant physical exam findings, and infant workup. The CDC and American Academy of Pediatrics (AAP) have published guidelines to assist providers with the diagnosis and management congenital syphilis [39,45]. Key points from these guidelines are as follows (Figure 3 and Table 1): 

(1) All infants born to mothers with a known perinatal syphilis infection or positive syphilis screening (combination treponemal and nontreponemal) during pregnancy or at delivery should undergo screening for congenital syphilis prior to discharge from the birth hospital. 

(2) The screening test for congenital syphilis is a nontreponemal (RPR) test. 

(3) Infants with physical exam findings concerning for congenital syphilis or RPR titer ≥4-fold maternal titer should have a workup (inclusive of cerebrospinal fluid/CSF analysis for cell count, protein, and CSF-VDRL; complete blood counts (CBC) and long-bone radiographs). These infants should receive 10 days of IV aqueous penicillin regardless of the workup results. 

(4) Infants with a normal physical exam, RPR titer <4-fold maternal titer, and mother with no treatment or treatment unknown, inadequate or initiated <30 days prior to delivery, should undergo a workup (inclusive of CSF analysis for cell count, protein, and CSF-VDRL; complete blood counts (CBC) and long-bone radiographs). If any part of the work-up is abnormal, uninterpretable, or incomplete, the infant should receive 10 days of IV aqueous penicillin. If the workup is normal, interpretable, complete, and followup for repeat RPR at 2 to 3 months is certain, the infant should receive a single dose of IM Benzathine penicillin. 

(5) Infants with a normal physical exam, RPR titer <4-fold maternal titer, and a mother who received treatment for perinatal syphilis infection that was adequate for maternal stage of infection, initiated >30 days before delivery with no concern for reinfection, no further workup is recommended. If followup for repeat RPR at 2 to 3 months after birth is uncertain, the baby can receive a single dose of IM Benzathine penicillin. No treatment is recommended if outpatient followup for repeat RPR in 2 to 3 months is assured.

(6) Infants with a normal physical exam, RPR titer <4-fold maternal titer and a mother who received adequate treatment for the maternal stage of infection prior to pregnancy with no concern for reinfection, no further workup or treatment is recommended for the infant. For babies with reactive RPR and for whom followup for repeat RPR at 2 to 3 months after birth is not certain, a single dose of IM Benzathine penicillin could be administered. 

(7) All infants with positive RPRs at birth or with a negative RPR but concern for possible incubating congenital syphilis should have followup RPR testing at 2 to 3 months after birth.

**Table 1:** Adapted evaluation and management guidelines based on CDC and AAP risk-stratification algorithms [15].

Scenario (CDC) Risk Category (AAP)	Clinical History and Examination	Evaluation	Treatment
Scenario 1, Category: Confirmed proven or highly probable congenital syphilis	Abnormal physical exam OR RPR titer ≥ 4-fold maternal titer	CSF analysis (cell count, protein, VDRL), CBC, Long bone radiographs Other *	Intravenous Penicillin G × 10 days (regardless of evaluation results)
Scenario 2, Category: Possible congenital syphilis	Normal physical exam AND RPR titer < 4-fold maternal titer AND Maternal treatment none/unknown/inadequate or initiated <30 days before delivery	CSF analysis cell count, protein, VDRL, CBC, long-bone radiographs	Penicillin G × 10 days (if evaluation is abnormal, uninterpretable, incomplete, or followup uncertain OR Intramuscular Benzathine Penicillin × 1 (if evaluation and followup certain)
Scenario 3, Category: less likely congenital syphilis	Normal physical exam AND RPR titer < 4-fold maternal titer AND Maternal treatment: adequate and initiated ≥30 days before delivery and no concern for reinfection	None	Intramuscular Benzathine Penicillin × 1 (if followup uncertain) OR No treatment with follow-up titers (followup certain)
Scenario 4, Category: unlikely congenital syphilis	Normal physical exam AND RPR titer < 4-fold maternal titer AND Maternal treatment adequate before pregnancy	None	No treatment with followup RPR titers (if infant RPR positive) OR Intramuscular Benzathine Penicillin × 1 (if followup uncertain)

Table adapted from Fang et al. [15] (https://creativecommons.org/licenses/by/4.0/, accessed on 20 May 2023). CSF, cerebral spinal fluid; CBC, complete blood count; RPR = rapid plasma reagin; VDRL, Venereal Disease Research Laboratory Test. * Other tests as clinically indicated (e.g., liver function tests, neuroimaging, ophthalmologic exam, and auditory brain stem response).

## 5. Public Health Interventions

**Figure 4:**
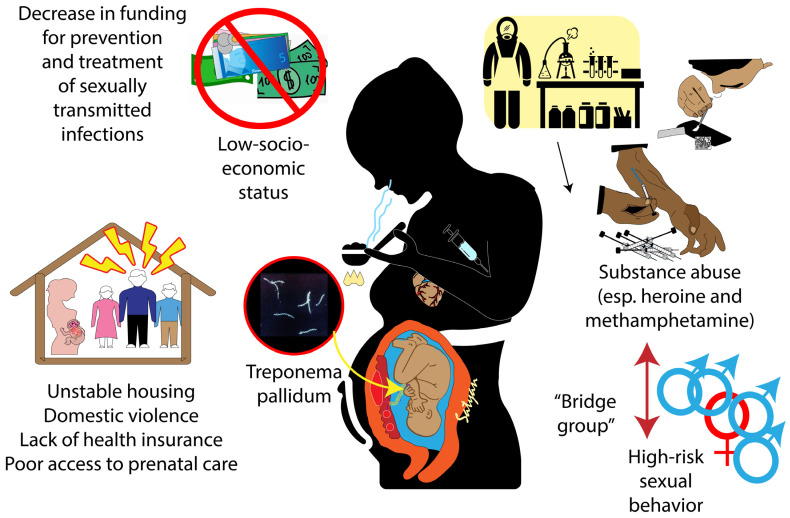
Risk factors for perinatal syphilis and congenital syphilis. System-level factors such as decreases in funding for prevention and treatment of sexually transmitted infections and poverty, community-level factors such as poor access to prenatal healthcare and individual level factors such as unstable housing, domestic violence, poor socioeconomic status, substance abuse (especially heroin and methamphetamine), and high-risk sexual behavior. Inset: dark-field microscopic view of Treponema pallidum subspecies pallidum. Copyright: Satyan Lakshminrusimha.

Geographical factors and socioeconomic determinants affect the exposure to substance use, STIs, and access to consistent and quality healthcare during pregnancy for adequate testing and treatment of perinatal syphilis (Figure 4 and Table 2) [50]. Financial stability plays a major role in the access to prenatal medical care for many disadvantaged persons. Unstable housing, language barriers, lack of insurance, and transportation are well-reported barriers to prenatal care [51,52,53,54]. 

**Substance abuse as an barrier to seek prenatal care services:** Substance use is a risk factor for perinatal syphilis as it increases the risk of transmission of STIs due to high-risk sexual behaviors including transactional and unprotected intercourse [23]. Moreover, syphilis might be transmitted from persons who use substances to those who do not (“bridge group”), thus increasing spread to persons not using illicit substances. This in turn leads to decreased awareness of infection and continued transmission. Persons that engage in substance abuse may be reluctant to seek out healthcare because of possible legal repercussions and associated stigma surrounding substance use [55]. Additionally, those that do utilize healthcare may underreport substance use and symptoms of syphilis infection when asked retrospectively. Secondly, substance use and its ties to risky sexual behavior (multiple sexual partners, transactional sex, and unprotected sex) [56,57] may increase STI transmission, subsequently increasing the incidence of congenital syphilis. Wide-scale implementation of needle exchange programs throughout the county have been shown to reduce risky sexual behavior. Combining STI services and harm-reduction interventions has proven successful in urban settings, such as New York City, [58] while prevention strategies are also at work in predominantly rural communities [59]. The effect of this strategy in a rural or remote settings has not been extensively researched and is, therefore, unknown. However, as the epidemics of substance use and congenital syphilis intersect, treating sexual health and drug use simultaneously is warranted. Harm reduction services, such as needle exchange programs, can be combined with sexual health education, outreach and testing of persons at risk of using substances [60]. Such programs have observed previous success in relaying persons at risk to secondary sources of treatment of substance use disorders and may be able to produce similar results for STI treatment [61]. The combination of these interventions also provides an opportunity for healthcare officials to test for STIs. Outreach programs including readily understandable educational material are crucial at this juncture to inform about programs for the prevention and management of perinatal syphilis and congenital syphilis [15]. Employers and local health departments alike must carefully consider the vulnerability of certain persons who are at risk (socioeconomically and otherwise disadvantaged) of perinatal syphilis, and, hence, congenital syphilis in their children, and provide proper access to early serological testing and prenatal treatment [4].

In LMICs, the factors contributing to increased transmission of syphilis may be different and multifactorial. In addition to factors reported from HICs, lack of awareness, overcrowding, working in environments with poor sanitation, lack of access to appropriate screening tests in remote rural areas, and lack of accurate reporting with underreporting of cases may need to be considered in LMICs [4]. 

**Widespread access to testing and treatment.** Screening algorithms with high sensitivity and specificity for accurate detection of syphilis are currently available. Both treponemal and nontreponemal serologic tests have a specificity of 98–100% and very high sensitivity approaching 100% [62]. A focus and commitment from public health departments and increasing awareness among healthcare providers are the cornerstone of achieving a reduction in rates of syphilis and congenital syphilis. Although an initial focus on persons who are at risk of acquiring and transmitting syphilis including HIV-infected persons, men who have sex with men, and persons who use substances especially among persons of color and socially disadvantaged populations is required [16], increasing screening to all sexually active persons in the communities with high-prevalence rates may potentially be an approach to maximize the effect of the intervention. Gaps in the current efforts in testing are listed in Table 3. 

Increased federal funding may allow mapping to identify geographical areas where widespread screening is urgently needed. Adequate funding will also allow an increase in the number of public health staff and use of point-of-care testing that can reach rural/remote areas [63]. The use of rapid tests alone in the absence of confirmatory tests may result in inaccurate surveillance. Therefore, having a well-linked health information system that links the testing, treatment, and followup is important in improving the quality of surveillance programs. Furthermore, this may provide timely information to the policy makers in public health. Introduction of mobile clinics for screening and testing for syphilis in addition to other STIs in populations with high prevalence of syphilis to test and treat would counter the “access to testing and treatment” barrier. This will allow point-of-care testing for syphilis. In addition to testing individuals, an outreach nurse could offer treatment with IM penicillin to those individuals who test positive. Furthermore, this point of contact could be utilized in developing an ongoing relationship with the pregnant person and connect them with additional services including mental health support and substance-use deaddiction programs. 

## 6. Conclusions

Congenital syphilis is a preventable infection that is increasing worldwide. It can cause death or involve multiple organ systems resulting in long-standing morbidity [2]. The resurgence of perinatal syphilis globally is due to a multitude of factors including substance abuse, low socioeconomic status, and inadequate public health infrastructure to reduce community transmission of perinatal syphilis [16]. While treatment after testing is a missed opportunity in HICs, socioeconomic barriers and access to testing and treatment are the major obstacles to congenital syphilis prevention in LMICs from currently available limited data. Public health interventions targeting early diagnosis of perinatal syphilis and timely management can potentially reduce the incidence of congenital syphilis. Furthermore, focused education of healthcare providers on early clinical recognition of syphilis and appropriate testing and management recommendations are warranted. Federal funding and media attention are crucial to curb this rising public health crisis. To summarize, there is an urgent need to strengthen public health funding and infrastructure, establish widespread surveillance testing, standardize surveillance metrics, prioritize STI prevention, improve availability and affordability of testing, integrate treatment guidelines into clinical practice, link the testing and treatment with the public health departments through health information systems streamlining case notification flow that allow appropriate documentation and reporting of cases, and ultimately the development of health policies to tackle this global crisis. 

## Figures and Tables

**Table 2:** Risk factors for perinatal syphilis infection.

Type of Risk Factor	Description
At individual level	High risk sexual behaviors Substance use Belonging to vulnerable groups either due to geographic location or race/ethnicity Poor health literacy, language barriers Inadequate prioritization of personal healthcare and poor utilization of available resources Stigma and fear of judgment Lack of health insurance
Community level	Inadequate access to healthcare Limited number of clinicians accepting Medicaid Inadequate medical knowledge on syphilis among clinicians and limited guidance Judgmental and stigmatizing approach Poor provision of sexual health education
System level	Poverty Structural and systemic racism Homelessness and housing insecurity Inadequate focus and resource allocation to rural and remote areas Lack of resources to ensure contact tracing and followup Lack of funding Inadequate public health infrastructure Integration of health information systems to provide information to health policy makers

**Table 3:** Gaps in the current efforts for syphilis testing.

Gaps in the current testing for perinatal syphilis
**Healthcare delivery: ** Isolated care systems with inadequate infrastructure Insufficient screening during pregnancy by clinicians Inadequately implemented screening protocols Complexity of syphilis screening and diagnosis Delay in testing Testing below par in areas of high prevalence of syphilis and at-risk populations Insufficient efforts for contact tracing and followup
**Pregnant person:** Missed prenatal visits and noncompliance with testing and treatment. Mistrust in healthcare and public health system Transiency of pregnant people and relationship instability Substance use Mental health issues Complexity of testing and followup

## Data Availability

Data presented in this study are available in this manuscript.

## References

[1] Penner J., Hernstadt H., Burns J.E., Randell P., Lyall H. (2021). Stop, think SCORTCH: Rethinking the traditional ‘TORCH’ screen in an era of re-emerging syphilis. Arch. Dis. Child..

[2] Force U.P.S.T. (2022). Screening for Syphilis Infection in Nonpregnant Adolescents and Adults: US Preventive Services Task Force Reaffirmation Recommendation Statement. JAMA.

[3] Salomè S., Cambriglia M.D., Scarano S.M., Capone E., Betts I., Pacella D., Sansone M., Mazzarelli L.L., Lo Vecchio A., Ranucci G. (2023). Congenital syphilis in the twenty-first century: An area-based study. Eur. J. Pediatr..

[4] Gilmour L.S., Walls T. (2023). Congenital Syphilis: A Review of Global Epidemiology. Clin. Microbiol. Rev..

[5] Paixao E.S., Ferreira A.J., Dos Santos I.O., Rodrigues L.C., Fiaccone R., Salvi L., de Oliveira G.L., Santana J.G., Cardoso A.M., Teles C. (2023). Mortality in children under 5 years of age with congenital syphilis in Brazil: A nationwide cohort study. PLoS Med..

[6] Hawkes S., Matin N., Broutet N., Low N. (2011). Effectiveness of interventions to improve screening for syphilis in pregnancy: A systematic review and meta-analysis. Lancet Infect. Dis..

[7] Knell R.J. (2004). Syphilis in renaissance Europe: Rapid evolution of an introduced sexually transmitted disease?. Proc. Biol. Sci..

[8] Pesapane F., Marcelli S., Nazzaro G. (2015). Hieronymi Fracastorii: The Italian scientist who described the “French disease”. An. Bras. Dermatol..

[9] Douglas J.M. (2009). Penicillin Treatment of Syphilis. JAMA.

[10] Kojima N., Klausner J.D. (2018). An Update on the Global Epidemiology of Syphilis. Curr. Epidemiol. Rep..

[11] Clement M.E., Hicks C.B. (2016). Syphilis on the Rise: What Went Wrong?. JAMA.

[12] Centers for Disease Control and Prevention Syphilis—CDC Detailed Fact Sheet. https://www.cdc.gov/std/syphilis/stdfact-syphilis-detailed.htm.

[13] Kiarie J., Mishra C.K., Temmerman M., Newman L. (2015). Accelerating the dual elimination of mother-to-child transmission of syphilis and HIV: Why now?. Int. J. Gynaecol. Obstet..

[14] WHO (2021). Global Progress Report on HIV, Viral Hepatitis and Sexually Transmitted Infections, 2021. https://www.who.int/publications/i/item/9789240027077.

[15] Fang J., Silva R.M., Tancredi D.J., Pinkerton K.E., Sankaran D. (2022). Examining associations in congenital syphilis infection and socioeconomic factors between California’s small-to-medium and large metro counties. J. Perinatol..

[16] Centers for Disease Control and Prevention (2023). Table 21. Congenital Syphilis—Reported Cases and Rates of Reported Cases by Year of Birth, by State/Territory* and Region in Alphabetical Order, United States, 2017–2021. https://www.cdc.gov/std/statistics/2021/tables/21.htm.

[17] Harville E.W., Giarratano G.P., Buekens P., Lang E., Wagman J. (2021). Congenital syphilis in East Baton Rouge parish, Louisiana: Providers’ and women’s perspectives. BMC Infect. Dis..

[18] Crowley J.S., Geller A.B., Vermund S.H., National Academies of Sciences, Engineering, and Medicine, Health and Medicine Division, Board on Population Health and Public Health Practice, Committee on Prevention and Control of Sexually Transmitted Infections in the United States (2021). Sexually Transmitted Infections: Adopting a Sexual Health Paradigm.

[19] Sankaran D., Lakshminrusimha S., Manja V. (2022). Methamphetamine: Burden, mechanism and impact on pregnancy, the fetus, and newborn. J. Perinatol..

[20] Kimball A., Torrone E., Miele K., Bachmann L., Thorpe P., Weinstock H., Bowen V. (2020). Missed Opportunities for Prevention of Congenital Syphilis—United States, 2018. Morb. Mortal. Wkly. Rep..

[21] Newman L., Kamb M., Hawkes S., Gomez G., Say L., Seuc A., Broutet N. (2013). Global estimates of syphilis in pregnancy and associated adverse outcomes: Analysis of multinational antenatal surveillance data. PLoS Med..

[22] Joseph Davey D., Shull H., Billings J., Wang D., Adachi K., Klausner J. (2016). Prevalence of Curable Sexually Transmitted Infections in Pregnant Women in Low- and Middle-Income Countries From 2010 to 2015: A Systematic Review. Sex. Transm. Dis..

[23] Coffin L.S., Newberry A., Hagan H., Cleland C.M., Des Jarlais D.C., Perlman D.C. (2010). Syphilis in drug users in low and middle income countries. Int. J. Drug Policy.

[24] Zheng Y., Yu Q., Lin Y., Zhou Y., Lan L., Yang S., Wu J. (2022). Global burden and trends of sexually transmitted infections from 1990 to 2019: An observational trend study. Lancet Infect. Dis..

[25] Fu L., Sun Y., Han M., Wang B., Xiao F., Zhou Y., Gao Y., Fitzpatrick T., Yuan T., Li P. (2022). Incidence Trends of Five Common Sexually Transmitted Infections Excluding HIV From 1990 to 2019 at the Global, Regional, and National Levels: Results From the Global Burden of Disease Study 2019. Front. Med..

[26] Kahn J.G., Jiwani A., Gomez G.B., Hawkes S.J., Chesson H.W., Broutet N., Kamb M.L., Newman L.M. (2014). The cost and cost-effectiveness of scaling up screening and treatment of syphilis in pregnancy: A model. PLoS ONE.

[27] Hawkes S.J., Gomez G.B., Broutet N. (2013). Early antenatal care: Does it make a difference to outcomes of pregnancy associated with syphilis? A systematic review and meta-analysis. PLoS ONE.

[28] Sunny M.P., Krishnan C., Abdulla P.S., Geeta M.G. (2022). Congenital syphilis: Need for intensification of antenatal screening and clinician awareness. Trop. Doct..

[29] Yue X., Gong X., Li J., Zhang J. (2021). Epidemiological trends and features of syphilis in China, 2014–2019. Chin. J. Dermatol..

[30] Gao J., Chen X., Yang M., Wu Y., Liang T., Li H., Xie W. (2023). Adverse pregnancy outcomes and associated risk factors among pregnant women with syphilis during 2013–2018 in Hunan, China. Front. Med..

[31] Wang H., Ying X., Lin D., Uwimana M.M.P., Zhang X. (2023). Towards the elimination of mother to child transmission of syphilis 2015–2020: Practice and progress in Zhejiang province, eastern China. BMC Pregnancy Childbirth.

[32] Qin J.B., Feng T.J., Yang T.B., Hong F.C., Lan L.N., Zhang C.L., Liu X.L., Yang Y.Z., Xiao S.Y., Tan H.Z. (2014). Synthesized prevention and control of one decade for mother-to-child transmission of syphilis and determinants associated with congenital syphilis and adverse pregnancy outcomes in Shenzhen, South China. Eur. J. Clin. Microbiol. Infect. Dis..

[33] Luo Z., Ding Y., Yuan J., Wu Q., Tian L., Zhang L., Li B., Mou J. (2021). Predictors of Seronegative Conversion After Centralized Management of Syphilis Patients in Shenzhen, China. Front. Public Health.

[34] de Brito Pinto T.K., da Cunha-Oliveira A., Sales-Moioli A.I.L., Dantas J.F., da Costa R.M.M., Silva Moura J.P., Gómez-Cantarino S., Valentim R.A.M. (2022). Clinical Protocols and Treatment Guidelines for the Management of Maternal and Congenital Syphilis in Brazil and Portugal: Analysis and Comparisons: A Narrative Review. Int. J. Environ. Res. Public Health.

[35] Pinto R., Valentim R., Fernandes da Silva L., Fontoura de Souza G., Góis Farias de Moura Santos Lima T., Pereira de Oliveira C.A., Marques Dos Santos M., Espinosa Miranda A., Cunha-Oliveira A., Kumar V. (2022). Use of Interrupted Time Series Analysis in Understanding the Course of the Congenital Syphilis Epidemic in Brazil. Lancet Reg. Health Am..

[36] Bailey H., Turkova A., Thorne C. (2017). Syphilis, hepatitis C and HIV in Eastern Europe. Curr. Opin. Infect. Dis..

[37] Benoit P., Tennenhouse L., Lapple A., Hill-Carroll G., Shaw S., Bullard J., Plourde P. (2022). Congenital syphilis re-emergence in Winnipeg, Manitoba. Can. Commun. Dis. Rep..

[38] Public Health Agency of Canada (2020). Syphilis in Canada: Technical Report on Epidemiological Trends, Determinants and Interventions.

[39] Kimberlin D.W., Barnett E.D., Lynfield R., Sawyer M.H., Committee on Infectious Diseases, American Academy of Pediatrics (2021). Red Book: 2021–2024 Report of the Committee on Infectious Diseases.

[40] Fang J., Partridge E., Bautista G.M., Sankaran D. (2022). Congenital Syphilis Epidemiology, Prevention, and Management in the United States: A 2022 Update. Cureus.

[41] (2017). Congenital Syphilis-CDC Fact Sheet. https://www.cdc.gov/std/syphilis/cong-syph-oct-2019.pdf.

[42] Lawrence R.M., Lawrence R.A. (2004). Breast milk and infection. Clin. Perinatol..

[43] O’Connor M., Kleinman S., Goff M. (2008). Syphilis in Pregnancy. J. Midwifery Women’s Health.

[44] Centers for Disease Control and Prevention (2023). Contrindications of Breastfeeding. https://www.cdc.gov/breastfeeding/breastfeeding-special-circumstances/contraindications-to-breastfeeding.html.

[45] Centers for Disease Control and Prevention (2021). Syphilis during Pregnancy. https://www.cdc.gov/std/treatment-guidelines/syphilis-pregnancy.htm.

[46] Alexander J.M., Sheffield J.S., Sanchez P.J., Mayfield J., Wendel G.D. (1999). Efficacy of treatment for syphilis in pregnancy. Obstet. Gynecol..

[47] Roberts C.P., Raich A., Stafylis C., Klausner J.D. (2019). Alternative Treatments for Syphilis During Pregnancy. Sex. Transm. Dis..

[48] Aleem S., Walker L.S., Hornik C.D., Smith M.J., Grotegut C.A., Weimer K.E. (2022). Severe congenital syphilis in the neonatal intensive care unit: A retrospective case series. Pediatr. Infect. Dis. J..

[49] Kim Y.H., Song J.H., Kim C.J., Yang E.M. (2017). Congenital Syphilis Presenting with Only Nephrotic Syndrome: Reemergence of a Forgotten Disease. J. Korean Med. Sci..

[50] Dombrowski K., Crawford D., Khan B., Tyler K. (2016). Current Rural Drug Use in the US Midwest. J. Drug Abus..

[51] California Department of Housing and Community Development (2012). Farmworkers. https://www.hcd.ca.gov/community-development/building-blocks/housing-needs/farmworkers.shtml.

[52] National Center for Farmworker Health, Inc. (2017). Maternal and Child Health. http://www.ncfh.org/maternal-and-child-health.html.

[53] Hetrick M. (2015). Medicaid and Migrant Farmworkers: Why the State Residency Requirement Presents a Significant Access Barrier and What States Should Do About it. Health Matrix J. Law Med..

[54] Chan E.Y.L., Smullin C., Clavijo S., Papp-Green M., Park E., Nelson M., Giarratano G., Wagman J.A. (2021). A qualitative assessment of structural barriers to prenatal care and congenital syphilis prevention in Kern County, California. PLoS ONE.

[55] Slutsker J.S., Hennessy R.R., Schillinger J.A. (2018). Factors contributing to congenital syphilis cases—New York City, 2010–2016. Morb. Mortal. Wkly. Rep..

[56] Vera A., Abramovitz D., Lozada R., Martinez G., Rangel M.G., Staines H., Patterson T.L., Strathdee S.A. (2012). Mujer Mas Segura (Safer Women): A combination prevention intervention to reduce sexual and injection risks among female sex workers who inject drugs. BMC Public Health.

[57] Smullin C., Wagman J., Mehta S., Klausner J.D. (2021). A Narrative Review of the Epidemiology of Congenital Syphilis in the United States From 1980 to 2019. Sex. Transm. Dis..

[58] Appel P.W., Warren B.E., Yu J., Rogers M., Harris B., Highsmith S., Davis C. (2017). Implementing substance abuse intervention services in New York City sexually transmitted disease clinics: Factors promoting interagency collaboration. J. Behav. Health Serv. Res..

[59] (2015). Youth Community Health Assessment of Resources and Trends (CHART) Project. https://www.co.fresno.ca.us/home/showdocument?id=20461.

[60] Reno H., Fox B., Highfill C., McKee A., Trolard A., Liang S.Y., Stoner B.P., Meyerson B.E. (2020). The Emerging Intersection Between Injection Drug Use and Early Syphilis in Nonurban Areas of Missouri, 2012–2018. J. Infect. Dis..

[61] Fernandes R.M., Cary M., Duarte G., Jesus G., Alarcão J., Torre C., Costa S., Costa J., Carneiro A.V. (2017). Effectiveness of needle and syringe Programmes in people who inject drugs–An overview of systematic reviews. BMC Public Health.

[62] Clement M.E., Hicks C.B. (2014). RPR and the serologic diagnosis of syphilis. JAMA.

[63] Castro A.R., Esfandiari J., Kumar S., Ashton M., Kikkert S.E., Park M.M., Ballard R.C. (2010). Novel point-of-care test for simultaneous detection of nontreponemal and treponemal antibodies in patients with syphilis. J. Clin. Microbiol..

